# Achievement of complete in vitro spermatogenesis in testicular tissues from prepubertal mice exposed to mono- or polychemotherapy

**DOI:** 10.1038/s41598-022-11286-6

**Published:** 2022-05-06

**Authors:** Marion Delessard, Laura Stalin, Aurélie Rives-Feraille, Laura Moutard, Justine Saulnier, Ludovic Dumont, Nathalie Rives, Christine Rondanino

**Affiliations:** INSERM, U1239, Team Adrenal and Gonadal Pathophysiology, Laboratory of Neuroendocrine Endocrine and Germinal Differentiation and Communication, Rouen University Hospital, Rouen Normandy University, 76000 Rouen, France

**Keywords:** Paediatric cancer, Reproductive biology, Infertility, Quality of life, Differentiation, Tissue engineering

## Abstract

The assessment of the impact of chemotherapies on in vitro spermatogenesis in experimental models is required before considering the application of this fertility restoration strategy to prepubertal boys who received these treatments before testicular tissue cryopreservation. The present work investigated the effects of exposure of prepubertal mice to mono- (vincristine or cyclophosphamide) and polychemotherapy (a combination of vincristine and cyclophosphamide) on the first wave of in vitro spermatogenesis. When testicular tissue exposed to monochemotherapy was preserved, polychemotherapy led to severe alterations of the seminiferous epithelium and increased apoptosis in prepubertal testes prior in vitro maturation, suggesting a potential additive gonadotoxic effect. These alterations were also found in the testicular tissues of polychemotherapy-treated mice after 30 days of organotypic culture and were associated with a reduction in the germ cell/Sertoli cell ratio. The different treatments neither altered the ability of spermatogonia to differentiate in vitro into spermatozoa nor the yield of in vitro spermatogenesis. However, more spermatozoa with morphological abnormalities and fragmented DNA were produced after administration of polychemotherapy. This work therefore shows for the first time the possibility to achieve a complete in vitro spermatogenesis after an in vivo exposure of mice to a mono- or polychemotherapy before meiotic entry.

## Introduction

Cancer treatment-related toxicities can generate late and long-term side effects in cured patients and infertility could be one of them^1–8^. For prepubertal patients who will receive gonadotoxic treatments, fertility preservation can be considered using testicular tissue freezing, which remains an experimental procedure^9–14^. Several approaches, mainly developed in animal models, could then be envisaged to mature the preserved testicular tissues in order to produce spermatozoa usable in assisted reproductive technology^10,12,14,15^. Among the fertility restoration strategies, in vitro spermatogenesis appears to be the most appropriate approach for patients with risks of testicular localization of tumour cells, for whom testicular autograft is not indicated. The three-dimensional (3D) cell culture and organotypic culture systems have been shown to support the differentiation of spermatogonia into spermatozoa in the mouse model^16–20^. Only the organotypic culture system allowed the production of murine spermatozoa from immature fresh or frozen/thawed testicular tissues, with a nuclear quality close to their in vivo counterparts and with the ability to generate a healthy and fertile offspring after intracytoplasmic sperm injection^16,20,21^.

The majority of prepubertal patients have received chemotherapy regimens, considered to be at low gonadotoxic risk, before the preservation of their testicular tissues^22,23^. Some studies have demonstrated that the administration of chemotherapy before puberty led to a decrease in the number of spermatogonia, especially when alkylating agents were included in the therapy regimen^24–26^. The depletion of the spermatogonial stem cell (SSC) pool could compromise the chances of fertility restoration. Since (i) the gonadotoxicity of chemotherapeutic agents is difficult to evaluate in humans because chemotherapy regimens associate several drugs and can be combined with radiotherapy and (ii) human prepubertal samples are scarce, the development of prepubertal animal models treated by chemotherapy is necessary to better understand the side effects of these treatments on in vitro spermatogenesis. So far, the in vitro maturation of prepubertal testicular tissues pre-exposed in vivo to chemotherapeutic agents and in which spermatogonia are the only germ cells has never been studied in animal models. The only attempts of in vitro maturation of testicular tissues recovered from prepubertal boys after the initiation of chemotherapy (treatment with a Berlin-Frankfurt-Munster-based chemotherapy regimen for acute lymphoblastic leukemia (ALL) prior to the testicular biopsy) were performed in a 3D methylcellulose culture system and led to the production of postmeiotic (acrosin +) cells after 15 weeks for one patient out of four^22^.

Cyclophosphamide (CYP) and vincristine (VCR), belonging respectively to the family of alkylating agents and spindle poisons, are used in the treatment of paediatric cancer including ALL, the most frequent cancer observed in prepubertal boys who benefitted from testicular tissue freezing^9,10,22,27^. In animal models, the few studies focusing on the impact of these molecules on prepubertal testicular tissues or spermatogonial cell lines have revealed their toxicity for spermatogonia. Indeed, a 24-h in vitro exposure of mouse prepubertal testes to CYP resulted in a significant loss of SSC associated with an increase in DNA double-stand breaks (DSBs)^28^. An increase in DNA DSBs has been also evidenced in a mouse spermatogonial C18-4 cell line after a 48-h exposure to CYP^29^. CYP treatment administered to prepubertal mice reduced the number of premeiotic cells 10 days after the last injection^30^. Moreover, the exposure of a rat spermatogonial cell line with SSC characteristics (GC-6spg) for 72 h to VCR resulted in the arrest of cell cycle and increased apoptosis without genotoxicity^31^. It should not be excluded that the proliferation and differentiation capacity of the spermatogonia remaining in the exposed testicular tissues may be disturbed. We recently reported that exposure of mice to low gonadotoxic doses of VCR or CYP before the initiation of meiosis led in adulthood respectively to (i) an altered progression of spermatogenesis associated with altered sperm morphology and nuclear quality or (ii) an increased production of spermatozoa with DNA damage^32^. Another study showed that the number of meiotic and postmeiotic cells was significantly reduced in CYP-treated immature mice 10 days after the end of the treatment^30^.

The aim of the present study was to assess, for the first time, the possibility to achieve a complete in vitro spermatogenesis from prepubertal mouse testicular tissues pre-exposed to a monochemotherapy (VCR or CYP) or a polychemotherapy (VCR+CYP) using the organotypic culture system. To this end, the impact of chemotherapeutic agents was analysed on fresh testicular tissues before in vitro culture and after the first wave of in vitro spermatogenesis.

## Results

### Short-term effect of prepubertal exposure to chemotherapy on testicular tissue before organotypic culture

**Figure 1:**
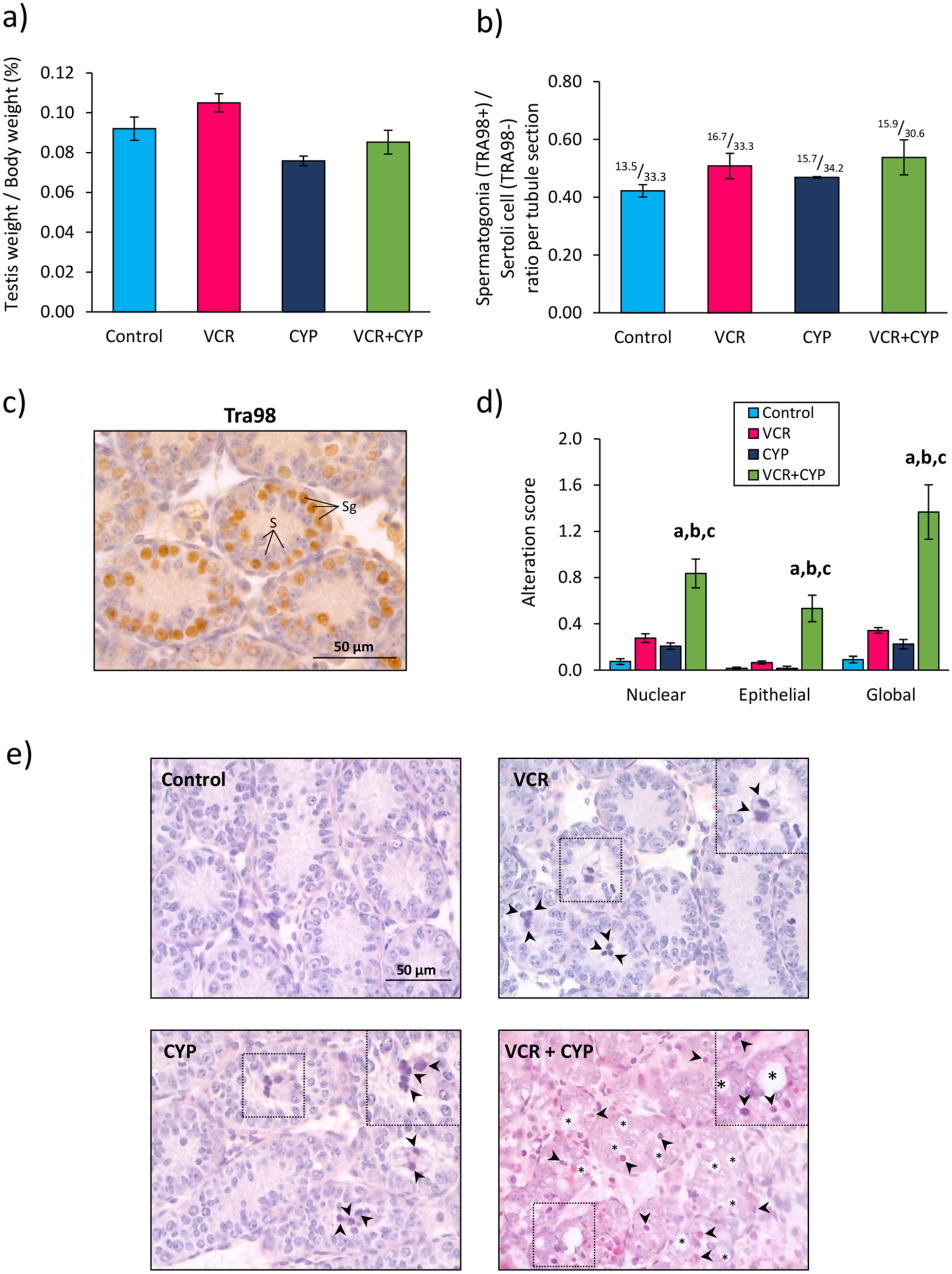
Impact of chemotherapy prepubertal exposure on mouse testis before organotypic culture. 5923915316674500Histograms representing (**a**) mean ratios of testicular weight to body weight and (**b**) mean ratios of spermatogonia to Sertoli cells per seminiferous tubule in 6 d*pp* mice treated with VCR, CYP or VCR+CYP or untreated (control) at 3 d*pp*. The ratio of the mean number of spermatogonia counted per seminiferous tubule to the mean number of Sertoli cells observed per tubule was specified above each bar. TRA98 immunostaining was performed to detect spermatogonia (**c**). Intratubular cells were identified as: S for Sertoli cells (irregular TRA98− blue nuclei) and Sg for spermatogonia (smooth spherical TRA98+ brown nuclei). Morphological alterations of the seminiferous tubules were assessed semi-quantitatively by calculating a global lesional score including nuclear and epithelial alterations (**d**). Representative microscopy images of Hemalun Eosin Saffron-stained 6 d*pp* mouse testicular tissues are shown at a ×400 magnification **(e)**. Panels on the upper right corner represent enlarged views of pyknotic nuclei () and vacuoles (*). Data are expressed as means ± SEM with n = 4 mice for all groups. A value of *P < 0.05 was considered statistically significant. ^a^*P* < 0.05 compared to control; ^b^*P* < 0.05 compared to VCR and ^c^*P* < 0.05 compared to CYP.

Prepubertal 3 days *post-partum* (d*pp*) mice received either an intraperitoneal injection of saline solution (NaCl 0.9% control group), vincristine (VCR group), cyclophosphamide (CYP group) or an association of vincristine and cyclophosphamide (VCR+CYP). Three days post-treatment, no significant difference in ratios of testis weight to body weight was observed in mice exposed to chemotherapy (VCR, CYP or VCR+CYP) compared to control group (Fig. 1a). Chemotherapy exposure did not affect the intratubular cell content since similar ratios between spermatogonia (TRA98+) and Sertoli cells (TRA98−) were observed in the different groups (Fig. 1b). The administration of VCR or CYP alone did not alter testicular tissue integrity in 6 d*pp* mice since no significant difference in the global lesional score (including nuclear and epithelial alterations) was evidenced with the control group (Fig. 1c). Although the lesional scores were similar between VCR, CYP and control groups, nuclear and epithelial alterations were noticed, including the presence of vacuoles and pyknotic nuclei, in the seminiferous tubules of VCR-treated and CYP-treated mice (Fig. 1d). Important nuclear alterations were observed in mice exposed to VCR+CYP (Fig. 1d). A significant threefold increase in the nuclear lesional score was found in the testes of mice exposed to both chemotherapeutical agents compared to the control group (0.84 *vs *0.08; *P* = 0.0012), VCR group (0.84 *vs *0.28; *P* = 0.0286) and CYP group (0.84 *vs *0.21; *P* = 0.0286) (Fig. 1c). Moreover, the presence of vacuoles (Fig. 1d) resulted in an at least fourfold higher epithelial lesional score in testicular tissues from VCR+CYP-treated mice than in control group (0.53 *vs *0.02; *P* = 0.0121), VCR (0.53 *vs *0.07; *P* = 0.0286) or CYP-treated mice (0.53 *vs *0.02; *P* = 0.0286) (Fig. 1c). A higher global lesional score was therefore measured after exposure to polychemotherapy, reflecting major alterations of testicular tissue integrity in comparison with mice exposed to VCR (1.37 *vs *0.34; *P* = 0.0286), CYP (1.37 *vs *0.22; *P* = 0.0286) or unexposed mice (1.37 *vs *0.09; *P* = 0.0014) (Fig. 1c).

### Cell proliferation, apoptosis and DNA double-strand breaks after chemotherapy

**Figure 2:**
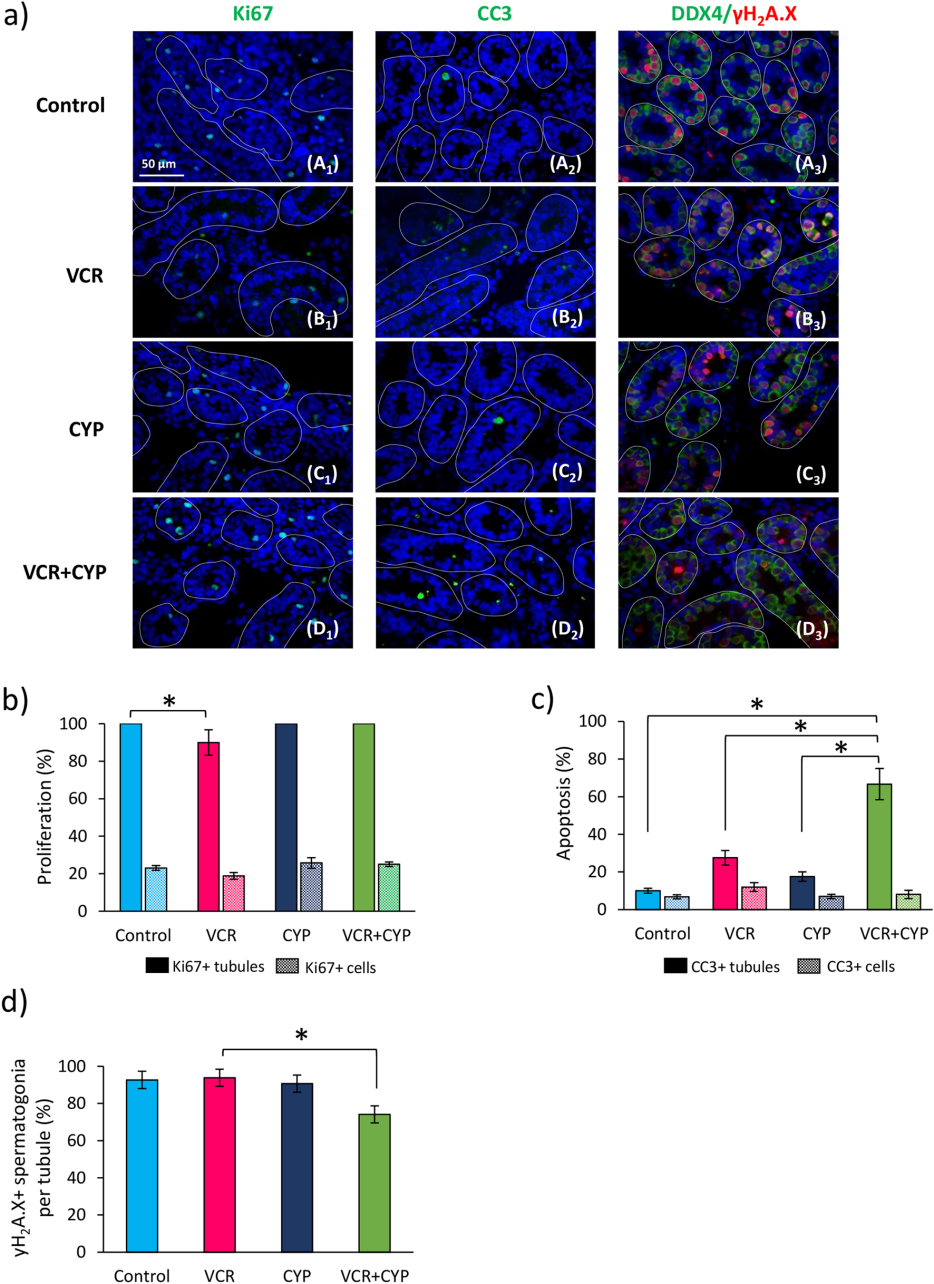
Impact of chemotherapy prepubertal exposure on intratubular cell before organotypic culture. **(a)** Representative images of Ki67 (A1-D1) and cleaved caspase-3 (A2-D2) immunofluorescence and combined detection of γH_2_A.X and DDX4 (A3-D3) in testicular tissues from 6 d*pp* control mice or VCR, CYP or VCR+CYP-treated mice. Testicular tissue sections were counterstained with DAPI (blue). **(b)** The percentage of intratubular cell proliferation (Ki67+ tubules in solid colour and Ki67+ cells in hatch colour) and **(c)** apoptosis (Caspase 3+ tubules in solid colour and cleaved caspase 3+ cells in hatch colour) were quantified for control, VCR, CYP and VCR+CYP groups. Double γH_2_A.X and DDX4 immunofluorescence was performed to determine the percentage of spermatogonia with DNA double-strand breaks for each condition **(d)**. Data are expressed as means ± SEM with n = 4 mice for all groups. A value of *P < 0.05 was considered statistically significant. Magnification ×400.

The percentage of seminiferous tubules with proliferating cells (Ki67+) (Fig. 2a A1–D1) decreased in 6 d*pp* mice after prepubertal exposure to VCR compared to control mice (90.00% *vs *100.00%; *P* < 0.0268) but no significant difference in the percentage of Ki67+ cells per tubule was found between these two groups (Fig. 2b). CYP and VCR+CYP treatments had no impact on intratubular cell proliferation (Fig. 2b). Cleaved caspase 3 immunostaining (CC3) (Fig. 2a A2–D2) showed a higher percentage of tubules with apoptotic cells in testes exposed to VCR+CYP compared to control (66.67% *vs *10.00%; *P* = 0.0014), VCR (66.67% *vs *27.50%; *P* = 0.0286), and CYP (66.67% *vs *17.50%; *P* = 0.0286) but no change in the proportion of apoptotic cells per tubule was observed (Fig. 2c). The administration of VCR or CYP alone did not lead to increased proportions of tubules containing apoptotic cells and of apoptotic cells per tubule in comparison to control mice (Fig. 2c). γH_2_A.X/DDX4 double immunostaining was used to visualize the presence of DNA DSBs in spermatogonia at 6 d*pp* (Fig. 2a A3–D3). A similar percentage of γH_2_A.X+ spermatogonia was found in the testes of VCR, CYP and VCR+CYP-exposed mice in comparison with unexposed mice (Fig. 2d). However, a decrease in the number of spermatogonia with DNA DSBs was observed in VCR+CYP group relative to VCR group (74.12% *vs *93.81%; *P* = 0.0286) (Fig. 2d).

### In vitro spermatogenesis in testicular tissues pre-exposed to chemotherapy

**Figure 3:**
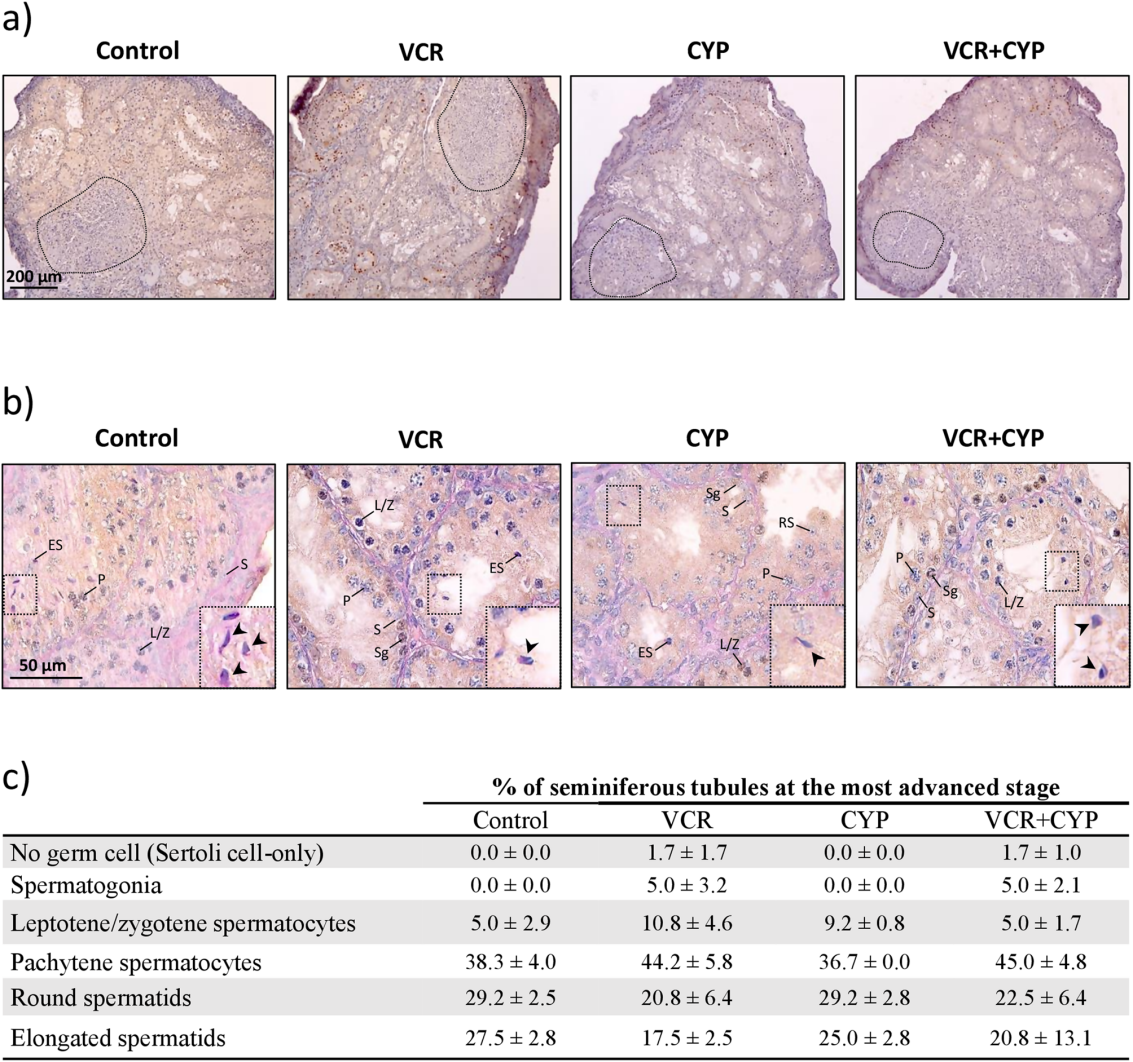
Progression of spermatogenesis following in vitro maturation of mouse testicular tissues treated by chemotherapy. (**a**) Microscopy images showing the general appearance of testicular tissue exposed to monochemotherapy (VCR or CYP), polychemotherapy (VCR+CYP) or unexposed (control) following 30 days of in vitro maturation. The necrotic area in cultured tissues has been delimited by dotted lines. (**b**) TRA98 immunostaining associated with Periodic Acid Schiff reaction was performed to visualize the progression of spermatogenesis after 30 days of organotypic culture, by detecting TRA98+ germ cells (spermatogonia, leptotene/zygotene and pachytene spermatocytes) and the pink-labelled acrosome (PAS+) of spermatids in control, CYP, VCR and VCR+CYP-treated mice. Intratubular cells were identified as: S for Sertoli cells (irregular TRA98- blue nuclei close to the basement membrane), Sg for spermatogonia (smooth spherical TRA98+ brown nuclei close to the basement membrane), L/Z for leptotene/zygotene spermatocytes (irregular spherical TRA98+ brown nuclei with condensed chromatin), P for pachytene spermatocytes (irregular spherical TRA98+ brown nuclei with highly condensed chromatin), RS for round spermatids (regular small round TRA98- blue nuclei and PAS+ acrosomal cap) or ES for elongated spermatids (elongated TRA98- blue nuclei with highly condensed chromatin and PAS+ acrosomal cap). Panels on the lower right corner represent a higher magnification of elongated spermatids (). (**c**) Percentage of seminiferous tubules at the most advanced differentiation stage of spermatogenesis after in vitro maturation of testicular tissues from mice exposed to VCR, CYP, VCR+CYP or unexposed (control) during the prepubertal period. Data are presented as means ± SEM with n = 4 testicular explants for each group. A value of *P < 0.05 was considered statistically significant.

Testicular tissues from 6 d*pp* mice treated with either VCR, CYP or VCR+CYP or untreated (control group) were matured in an organotypic culture system for 30 days, time required to achieve the first wave of spermatogenesis. As shown in our previous studies, a necrotic area was observed at the point of inclusion of the cultured fragments, with no obvious difference between control, VCR, CYP and VCR+CYP groups (Fig. 3a). The progression of spermatogenesis was assessed by determining the percentage of seminiferous tubules containing the most advanced type of germ cells (spermatogonia, leptotene/zygotene spermatocyte, pachytene spermatocyte, round spermatid or elongated spermatid, ie containing at least one of these germ cell types) and located outside the necrotic region (Fig. 3). The mono- and polychemotherapy treatments did not alter the ability of spermatogonia to differentiate in vitro since more than 17% of the seminiferous tubules contained elongated spermatids in VCR, CYP and VCR+CYP groups (Fig. 3b, c). Moreover, no significant difference in the proportion of tubules at the most advanced stage has been highlighted in all the conditions, thereby showing that chemotherapy treatments did not disturb the progression of spermatogenesis (Fig. 3c). However, although no significant difference was found, it should be noted that tubules with no germ cell were observed in cultured testicular tissues exposed to VCR or VCR+CYP, as well as an arrest of spermatogenesis at the spermatogonia stage in 5% of seminiferous tubules (Fig. 3c).

### Germ cell content, cell proliferation, apoptosis and DNA double-strand breaks after chemotherapy exposure and organotypic culture

**Figure 4:**
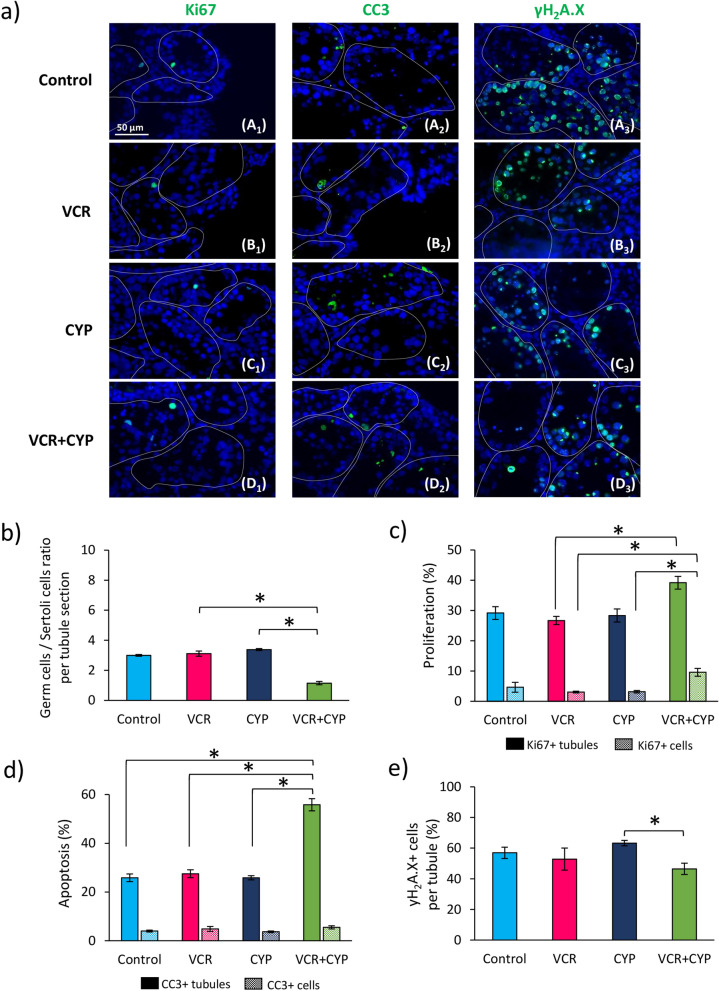
Impact of chemotherapy prepubertal exposure on intratubular cell following 30 days of in vitro maturation. (**a**) Representative images of Ki67 (A1–D1), cleaved caspase-3 (A2–D2) and γH_2_A.X (A3–D3) immunofluorescence in testicular tissues from control, VCR, CYP or VCR+CYP-treated mice after 30 days of culture. Testicular tissue sections were counterstained with DAPI (blue). (**b**) Histogram representing the mean ratio of germ cells to Sertoli cells per seminiferous tubule in cultured testicular fragments for each group. Ki67, cleaved caspase-3 and γH_2_A.X immunodetection was performed to determine (**c**) the percentage of intratubular cell proliferation (Ki67+ tubules in solid colour and Ki67+ cells in hatch colour), (**d**) apoptosis (Caspase 3+ tubules in solid colour and cleaved caspase 3+ cells in hatch colour) and (**e**) intratubular cells with DNA double-strand breaks for each condition. The results are presented as means ± SEM with n = 4 testicular explants for all groups. A value of *P < 0.05 was considered statistically significant. Magnification ×400.

The germ cells to Sertoli cells ratio was determined on TRA98-PAS-stained tissue sections. After 30 days of culture, a decreased germ cells to Sertoli cells ratio was observed in testes of mice exposed to VCR+CYP relative to those exposed to VCR (1.15 *vs *3.11; *P* = 0.0286) or CYP (1.15 *vs *3.38; *P* = 0.0286) (Fig. 4b). However, the ratios measured in the testicular tissues of chemotherapy-treated mice (VCR, CYP or VCR+CYP) were not significantly different from the control group (Fig. 4b). Ki67 immunostaining (Fig. 4a A1–D1) showed an increase in the percentage of tubules with proliferating cells in VCR+CYP group compared to VCR group (39.17% *vs *26.67%; *P* = 0.0286) but no difference was evidenced with control and CYP groups (Fig. 4c). Moreover, a threefold increase in the proportion of Ki67+ cells per tubule was found in testicular tissues exposed to VCR+CYP compared to testes exposed to VCR (9.55% *vs *3.03%; *P* = 0.0286) or CYP (9.55% *vs *3.16%; *P* = 0.0286) (Fig. 4c). A higher percentage of tubules with apoptotic cells was found after 30 days of culture in VCR+CYP-treated mice compared to mice in control group (55.83% *vs *25.83%; *P* = 0.0224), or exposed to VCR (55.83% *vs *27.50%; *P* = 0.0286) or CYP (55.83% *vs *25.83%; *P* = 0.0286) but no change in the proportion of apoptotic cells per tubule was evidenced (Fig. 4a A2–D2, d). The administration of VCR or CYP alone did not lead to an increase in the proportions of tubules containing apoptotic cells and of apoptotic cells per tubule in comparison to controls (Fig. 4d). Finally, less γH_2_A.X+ intratubular cells were observed in VCR+CYP-exposed mice compared to CYP-exposed mice whereas a similar proportion of intratubular cells with DNA DSBs was observed in VCR, CYP and VCR+CYP-exposed mice in comparison to control mice (Fig. 4a A3–D3, e).

### Quality of in vitro produced spermatozoa in chemotherapy-exposed tissues

**Figure 5:**
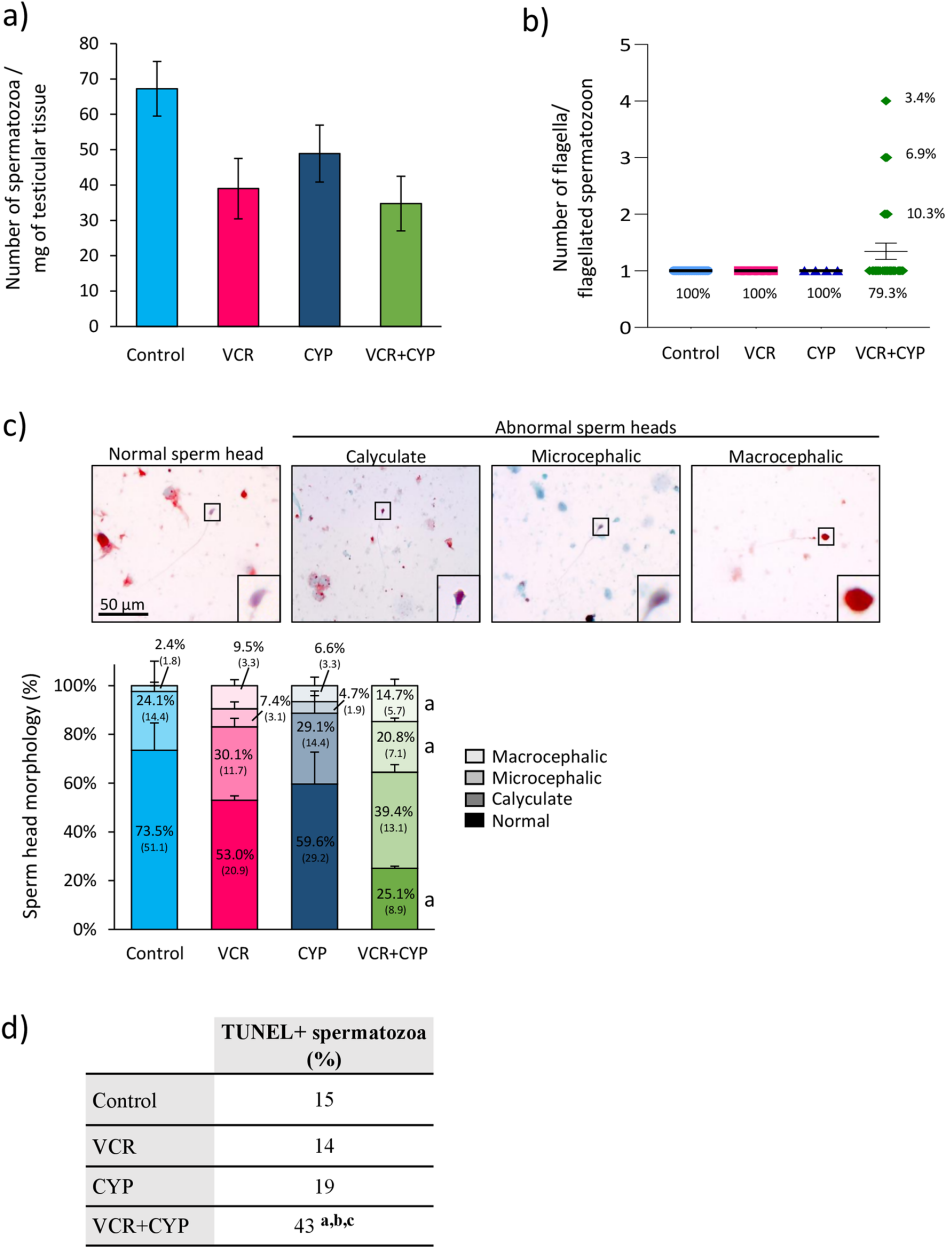
Impact of chemotherapy prepubertal exposure on in vitro produced spermatozoa. (**a**) Histogram representing the mean number of spermatozoa per milligram of tissue produced after in vitro maturation of testicular tissues from mice exposed to monochemotherapy (VCR or CYP), polychemotherapy (VCR+CYP) or unexposed (control) during the prepubertal period. (**b**) Percentage of spermatozoa with one or multiple (2, 3 or 4) flagella among the flagellated spermatozoa recovered from testicular tissues of control, VCR, CYP or VCR+CYP-treated mice after 30 days of culture. (**c**) Histogram representing the proportion of in vitro produced spermatozoa with normal or abnormal heads in testicular tissues exposed to VCR, CYP, VCR+CYP or unexposed (control). Sperm heads were assigned to two categories based on morphological observations: normal (elongated and hook-shaped) and abnormal^33^. Abnormal heads included calyculate, microcephalic (small rounded head) and macrocephalic (large rounded head). The mean number of spermatozoa counted per milligram of tissue is indicated in brackets for each category of sperm head morphology. (**d**) Percentage of in vitro produced spermatozoa with fragmented DNA (TUNEL+) in testicular tissues from control, VCR, CYP or VCR+CYP-treated mice. For each condition, hundred spermatozoa were examined. The results are presented as means ± SEM with n = 6 pooled cultured testes per group. ^a^*P* < 0.05 compared to control; ^b^*P* < 0.05 compared to VCR and ^c^*P* < 0.05 compared to CYP.

Sperm yield was determined and sperm morphology was assessed after Shorr staining. No significant difference in the mean number of spermatozoa per milligram of in vitro matured tissues was evidenced between controls, mono- and polychemotherapy-exposed groups (Fig. 5a). Moreover, no significant difference in the proportion of monoflagellated spermatozoa was found between all the groups (Fig. 5b). However, spermatozoa with 2 flagella (10.3%), 3 flagella (6.9%) and 4 flagella (3.4%) were detected in testicular tissues exposed to VCR+CYP whereas no multiflagellated spermatozoa were observed in control, VCR and CYP groups (Fig. 5b). A similar proportion of spermatozoa presenting a normal head was found in control, VCR and CYP groups (Fig. 5c). However, a lesser proportion of spermatozoa with a normal head was produced in testicular tissues exposed to VCR+CYP relative to controls (25.07% *vs *73.52%; *P* = 0.0276). Indeed, the proportion of microcephalic and macrocephalic spermatozoa (20.76% *vs* 2.41% and 14.75% *vs *0.00%; *P* = 0.0231 and *P* = 0.0285, respectively) was higher in VCR+CYP-exposed tissues than in controls (Fig. 5c). Moreover, terminal deoxynucleotidyl transferase mediated dUTP nick end labeling (TUNEL) analyses revealed a > twofold increase in the proportion of spermatozoa with fragmented DNA in VCR+CYP group compared to control (43% *vs *15%; *P* < 0.0001), VCR (43% *vs *14%; *P* < 0.0001) and CYP groups (43% *vs *19%; *P* = 0.0002) (Fig. 5d).

## Discussion

The present study investigated the in vitro maturation of prepubertal testicular tissue from mice exposed in vivo to drugs used in induction and consolidation chemotherapy in leukaemia: vincristine (VCR), cyclophosphamide (CYP) or a VCR+CYP combination. In our mouse model, we show for the first time that VCR, CYP and VCR+CYP did not alter the ability of spermatogonia to differentiate in vitro into spermatozoa. However, VCR+CYP administration altered sperm morphology and nuclear quality.

The development of animal models treated by chemotherapy is indeed necessary to better understand the side effects of cancer treatments administered before puberty on male gonads and the possibility to restore fertility from exposed testicular tissues. Prior to in vitro maturation using organotypic culture, the testicular tissues from 6 d*pp* mice exposed to chemotherapy at 3 d*pp* were first analysed. At this age, the only germ cells present in the testis are spermatogonia, known to be particularly sensitive to chemotherapy because of their high mitotic activity^34^. A significant loss of SSC associated with increased apoptosis have been reported in mouse prepubertal testis after a 24-h in vitro exposure to CYP^28^. Moreover, a 24-h in vitro exposure to alkylating agents such as cisplatin or carboplatin induced a loss of SSC and a reduction in proliferating germ cells in mouse prepubertal testis^35,36^ and in human prepubertal testis^37^ one week and 96 h after exposure, respectively. A rise in DNA DSBs has been also evidenced in a mouse spermatogonial cell line (C18-4 cells) 48 h after CYP exposure^29^. A reduction in testicular weight, in the number of germ cells, Sertoli and peritubular cells was recently reported in immature mice 10 days post-CYP treatment^30^. The cytotoxicity of VCR also led to a decreased viability and an increased apoptosis of a rat spermatogonial cell line with SSC characteristics after a 72-h exposure^31^. In our study, the ratio of testis weight to body weight, germ cell content, testicular tissue integrity and spermatogonial DNA integrity were preserved 3 days after the in vivo administration of clinically relevant-concentrations of VCR or CYP. In addition, the proliferation/apoptosis balance was not altered after CYP exposure, and only a slight decrease in the proportion of tubules with proliferating cells was found in VCR-treated mice compared to control mice. VCR, a spindle poison, is indeed known to inhibit microtubule polymerization, cause cell cycle arrest and disrupt cell proliferation^38^. The discrepancy between our results and the data reported in the literature may be explained by the difference in experimental designs. Indeed, in order to mimic the situation encountered in the clinics, we exposed prepubertal mice (before the initiation of meiosis) to chemotherapy whereas in most studies prepubertal testes or testicular cells were exposed in vitro to these drugs. This direct contact is likely the cause of greater damaging effects. Moreover, the previously reported gonadotoxicity of CYP in immature mice as early as 10 days post-treatment is likely the consequence of the repeated injection of this drug and of the higher dose used^30^.

We also report for the first time that prepubertal exposure to VCR or CYP did not interfere with the completion of in vitro spermatogenesis. Neither disturbance in the proliferation/apoptosis balance and germ cell content nor increase in DNA DSBs were observed in VCR- or CYP-treated mice compared to controls. A complete in vitro spermatogenesis was achieved in the testicular tissues of mice treated with monochemotherapy, with the presence of elongated spermatids in 17.5% to 25% of seminiferous tubules and the production of 39 to 49 spermatozoa per milligram of testicular tissue.

So far, only three studies have reported the in vitro production of spermatids/postmeiotic cells in cultures of monochemotherapy-exposed testicular tissues^30,36,39^. In the first study, 7 d*pp* mice were treated with busulfan and the testicular cells isolated at 17 d*pp* were cultured in a 3D methylcellulose culture system, with the generation of sperm-like cells after 4 weeks^39^. However, primary spermatocytes are already present in 17 d*pp* mouse testes^34^. It is therefore not excluded that the sperm-like cells could originate from the differentiation of spermatocytes rather than spermatogonia. Here, prepubertal mice were treated by chemotherapy at 3 d*pp* and testicular tissues were cultured starting from 6 d*pp*, i.e. before the initiation of meiosis, in order to ascertain that a complete in vitro spermatogenesis can be obtained from spermatogonia of chemotherapy-exposed animals. In the second study, 5 d*pp* mouse testes were exposed in vitro for one day to either cisplatin or doxorubicin and cultured on a polycarbonate membrane, with the generation of elongated spermatids after 4 and 8 weeks^36^. This experimental approach does not reproduce the fertility restoration procedure that could be implemented for boys with cancer i.e. the in vitro maturation of prepubertal testicular tissues after an in vivo exposure to chemotherapy drugs. In the last study, 9–10 d*pp* mice were treated with CYP once a week for 3 weeks and the spermatogonial cells isolated after treatment were cultured in a 3D methylcellulose culture system, with a differentiation up to the meiotic/postmeiotic (acrosin+) stage after 4–5 weeks, albeit not up to the spermatozoa stage^30^.

Since prepubertal boys often receive polychemotherapy in clinical practice, notably a combination of VCR and CYP in cases of acute leukemia, we next determined the effect of a coadministration of these molecules on the immature testis 3 days post-exposure and after a 30-day organotypic culture. Testes from 6 d*pp* VCR+CYP-treated mice displayed a 3- and fourfold increase in nuclear and epithelial alterations respectively compared to control mice and to mice exposed to VCR or CYP alone. Moreover, the percentage of tubules with apoptotic cells was at least twofold higher after VCR+CYP exposure than in control and after exposure to VCR or CYP. These results suggest that the administration of VCR+CYP may have additive gonadotoxic effects. It has been previously shown in men that vinca-alkaloids such as VCR can indeed have additive effects leading to prolonged azoospermia when given with alkylating agents such as CYP^40^. However, no change in the spermatogonia to Sertoli cells ratio was observed 3 days after VCR+CYP exposure. In addition, the proportion of γH_2_A.X+ spermatogonia was lower in VCR+CYP-exposed mice than in VCR-exposed mice. Histone H_2_A.X is phosphorylated (γH_2_A.X) in response to DNA DSBs induction and is commonly used as a marker for DNA damage^41^. However, during spermatogenesis, the formation of DNA DSBs is a physiological and programmed mechanism required for chromatin reorganization along mitotic and meiotic processes and spermatid differentiation. It has been reported that γH_2_A.X was present in all spermatogonia subtypes (A, intermediate and B) and was correlated with spermatogonial differentiation^42^. Therefore, the decreased percentage of γH_2_A.X+ spermatogonia observed in the testes of VCR+CYP-exposed animals could reflect an alteration of spermatogonial differentiation. The tissue alterations found in the testes of VCR+CYP-treated mice before organotypic culture did not prevent the progression of in vitro spermatogenesis up to the production of spermatozoa in 30-day organotypic cultures. Apoptosis was however noticed in almost 55% of the seminiferous tubules in in vitro matured tissues from VCR+CYP-treated mice. The increased apoptosis in cultured testicular tissues associated with the altered spermatogonial differentiation before in vitro culture could be responsible for the decrease in germ cell content. Since γH_2_A.X accompanies germ cell development, a loss of germ cells inevitably implies a decrease in γH_2_A.X+ germ cells. The slight increase in proliferating cells per tubule observed at the end of in vitro maturation could be a compensatory mechanism against germ cell depletion. As previously shown^20^, most of the spermatozoa produced in 30-day cultures of unexposed testicular tissues contained unfragmented DNA. Whereas most of in vitro produced spermatozoa contained an intact DNA after VCR or CYP exposure, 43% of them showed DNA fragmentation in the VCR+CYP group. Moreover, microcephalic and macrocephalic spermatozoa as well as multiflagellated spermatozoa were produced in greater number in polychemotherapy-exposed tissues. The production of double-tailed spermatozoa has been previously reported in CYP-treated adult rats^43^. In men, large-headed multiflagellar spermatozoa were associated with a high rate of aneuploidy, probably the result of chromosome nondisjunction and/or cytokinesis defects during meiotic divisions^44^. The transcription of key genes involved in these processes such as Aurora kinase family genes, BIRC5, CDCA8, BUB1^44,45^ could also be affected by CYP-induced adducts. Moreover, as a spindle poison, VCR probably disturbs chromosome segregation during germ cell divisions. In order to produce spermatozoa devoid of alterations, it may thus be necessary to perform more rounds of in vitro spermatogenesis. An organotypic culture exhaustion characterized by a cessation of spermatogenesis after 45 days of culture has been previously reported in the mouse model^16^. In human, spermatogonia hardly survive over a long period of culture and a significant germ cell loss is observed after 64 days of organotypic culture^46^. In the future, it would therefore be preferable to use a microfluidic device for long-term cultures and for a human application, as this method allowed the maintenance of in vitro spermatogenesis for more than 6 months in the mouse model^47^.

The present study shows for the first time the possibility to obtain a complete in vitro spermatogenesis in cultures of mouse prepubertal testicular tissues pre-exposed in vivo to VCR or CYP alone or to VCR+CYP. These results may be promising for clinical application in patients with risks of cancer recurrence after autograft. However, an in-depth evaluation of the nuclear quality of in vitro produced spermatozoa from chemotherapy-exposed tissues will have to be performed in order to ensure that they can be used in assisted reproductive technology. In addition, whether spermatozoa can be produced in cultures of frozen/thawed VCR, CYP or VCR+CYP-exposed testes will have to be determined.

## Methods

### Exposure of prepubertal mice to chemotherapy and samples collection

All the animal procedures were approved by the Institutional Animal Care and Use Committee of Rouen Normandy University (protocol APAFiS #18,208) and were carried out in accordance with relevant guidelines, regulations, and recommendations, including the ARRIVE guidelines. CD-1 mice (Charles River Laboratories, L’Arbresle, France) were housed on a 12 h light:12 h dark cycle, with food and water ad libitum. Prepubertal 3 d*pp* mice received either saline solution (control group), 100 µg/kg vincristine (ONCOVIN, Teva Santé, Paris, France) (VCR group), 15 mg/kg cyclophosphamide (ENDOXAN, Sanofi, Paris, France) (CYP group), or a combination of 100 µg/kg vincristine and 15 mg/kg cyclophosphamide (VCR+CYP group). The treatments were administered intraperitoneally in a single dose. The drugs used are the same as those employed in paediatric cancer treatment, during induction and consolidation chemotherapy in leukemia treatment, and the doses have been adapted to the murine model with a standard factor to interspecies doses conversion, as recommended by the Food and Drug Administration^27,48^. Throughout the study, analyses were performed by assessors blind to the treatment.

Testes from 6 d*pp* mice were obtained after euthanasia by decapitation and rinsed in α-MEM (Gibco by Life Technologies, Saint-Aubin, France) at 4 °C. The tunica albuginea was removed with needles and testicular tissues were then either cultured directly or fixed in Bouin’s solution or in 4% paraformaldehyde (PFA, Sigma-Aldrich, Saint-Quentin Fallavier, France) to analyse tissues before organotypic culture.

In total, 80 testes (32 testes from 6 d*pp* mice + 48 in vitro cultured testes) from 40 mice were used in this study (Supplementary Fig. 1).

### Organotypic cultures

Tissues were cultured at a gas–liquid interphase according to the previously published in vitro organ culture technique^16,18^. Briefly, 6 d*pp* mouse testes were cut into four fragments and partially inserted into a pair of 1.5% (w/v) agarose gels half soaked in medium. The culture medium was composed of α-MEM supplemented with 10% (v/v) KnockOut Serum Replacement (Gibco by Life Technologies) and 5 µg/mL gentamicin (Sigma-Aldrich). The medium was replaced every 4 days and a supplementation with 10^–6^ M retinol (Sigma-Aldrich) was performed every other time. Tissues were cultured under 5% CO_2_–95% air at 34 °C for 30 days. A total of 192 fragments (from 48 different testes) were maintained in vitro in this study (Supplementary Fig. 1).

### Histological analyses

Testicular tissues from 6 d*pp* mice and cultured explants were fixed in Bouin’s solution for 2 h at room temperature (RT). They were then dehydrated in graded series of ethanol and xylene in the Citadel 2000 tissue processor (Shandon, Cheshire, UK) and embedded in paraffin. Tissue Sections (3 µm thick) were cut using a microtome (JungRM 2035; Leica Microsystems) and mounted on Polysine slides (Thermo Fischer Scientific, Saint-Aubin, France). Tissue sections were deparaffinized in xylene and rehydrated with decreasing concentrations of ethanol.

Hemalun eosin saffron (HES) staining was performed to visualize tissue structural integrity and assign a global lesional score on a scale from 0 to 10 (0–5 for epithelial integrity and 0–5 for nuclear alteration, with 0 representing the complete absence of alteration and 5 representing the most important damage)^49^.

### Immunohistochemical analyses

TRA98 immunostaining was performed to discriminate between Sertoli cells (TRA98-) and spermatogonia, leptotene/zygotene and pachytene spermatocytes (TRA98 +) and to determine the germ cells to Sertoli cells ratio per tubule. TRA98 staining was associated with Periodic Acid Schiff (PAS) reaction (RAL diagnostic, Martillac, France) to detect the pink-labelled acrosome of spermatids after 30 days of in vitro maturation. Tissue sections were first incubated with a peroxidase-blocking solution (HP block; Dako REAL, Les Ulis, France) for 5 min followed by ultra-V blocking solution (Thermo Fisher Scientific) for 5 min at RT to inactivate endogenous peroxidases and prevent non-specific antibody binding, respectively. Rat anti-TRA98 antibodies (1:200; ab82527; Abcam, Paris, France) were then added and incubated for 30 min at RT. A negative control was performed with rat pre-immune IgGs (SC-2026, Santa Cruz Biotechnology, Heidelberg, Germany). After two washes in phosphate buffer saline (PBS) for 5 min, TRA98 was detected with a biotinylated rabbit anti-rat secondary antibody (1:200; ab6733; Abcam). A biotinylated goat anti-rabbit tertiary antibody (1:200; ab6720; Abcam) was incubated for 30 min after two 5-min PBS rinses. Specific staining was achieved by incubation with a horseradish peroxidase-conjugated streptavidin (TS-060-QPH, Thermo Fisher Scientific) for 5 min followed by an incubation with 3,3′-diaminobenzidine (1:50, Thermo Fischer Scientific) for 1 min at RT. Finally, tissue sections were stained with PAS. For each fragment or testis of each group, 30 cross-sectioned tubules (located outside the necrotic area) in 2 sections separated by at least 35 µm were analysed to obtain a global assessment of the tissue. Analyses were conducted with a light microscope (DM4000B, Leica Microsystems) equipped with a Leica Application Suite software.

Ki67, CC3 and γH_2_A.X immunofluorescence staining was performed to assess intratubular cell proliferation, apoptosis and DNA DSBs, respectively. The combined detection of γH_2_A.X and DDX4 allowed the identification of spermatogonia with DNA DSBs in tissues from 6 d*pp* mice. Prepubertal testes and cultured explants were fixed with PFA for 2 h at RT and the slides were prepared as previously described. After dewaxing and rehydration, tissues sections were washed in Tris buffer containing 0.05% (v/v) Tween-20 (TBST) for 3 min at RT and were boiled in 10 mM citrate buffer pH 6.0 (Diapath, Martinengo, Italy) for 40 min at 96 °C. They were cooled for 20 min at RT and rinsed in water for 5 min. A permeabilization step with 0.01% (v/v) Triton X-100 (Sigma-Aldrich) at RT for 15 min was performed for Ki67 staining only. Non-specific sites were blocked with 5% (w/v) bovine serum albumin (BSA, Sigma-Aldrich) and 5% (v/v) horse serum (Sigma-Aldrich) for Ki67 and CC3 staining, and with 20% (v/v) goat serum and 5% (w/v) BSA for γH_2_A.X and γH_2_A.X/DDX4 staining. Slides were then incubated with primary antibodies, rinsed thrice in TBST and incubated with secondary antibodies (Table 1). Sections were washed, dehydrated with ethanol and mounted in Vectashield with DAPI (4',6-diamidino-2-phenylindole). For each fragment or testis of each group, 30 cross-sectioned tubules (located outside the necrotic area) in 2 sections separated by at least 35 µm were analysed with a DM4000B light microscope (Leica Microsystems). The percentage of Ki67+ and CC3+ tubules was determined by dividing the number of tubules containing at least one positive cell by the number of tubules examined. When tubules were Ki67+ or CC3+, the number of positive cells was divided by the number of intratubular cells to obtain the percentage of positive cells per positive tubule. The percentage of γH_2_A.X+ spermatogonia per tubule at 6 d*pp* was calculated by dividing the number of γH_2_A.X+ DDX4+ cells by the number of DDX4+ cells. After 30 days of culture, the percentage of γH_2_A.X+ cells per tubule was measured by dividing the number of γH_2_A.X+ cells by the number of intratubular cells.

**Table 1:** Details of all the antibodies and conditions for immunofluorescence staining.

Primary antibody	Dilution	Supplier's reference	Incubation time	Secondary antibody	Dilution	Supplier's reference	Incubation time
**Ki67** (rabbit monoclonal)	1:500	Ab16667, Abcam, Paris, France	overnight (4 °C)	ALEXA FLUOR 488-conjugated anti-rabbit	1:200	Ab150077, Abcam, Paris, France	60 min (RT)
**Cleaved caspase3** (rabbit polyclonal)	1:200	Ab49822, Abcam, Paris, France	90 min (RT)	ALEXA FLUOR 488-conjugated anti-rabbit	1:200	Ab150077, Abcam, Paris, France	60 min (RT)
**γH**_**2**_**A.X** (mouse monoclonal)	1:200	JBW301 clone, Merck, Darmstadt, Germany	60 min (RT)	ALEXA FLUOR 488-conjugated anti-mouse	1:200	Ab150113, Abcam, Paris, France	60 min (RT)
co-immunostaining of **γH**_**2**_**A.X** (mouse monoclonal) and **DDX4** (rabbit polyclonal)	1:200 1:800	JBW301 clone, Merck, Darmstadt, Germany Ab13840, Abcam, Paris, France	overnight (4 °C)	biotinylated goat anti-mouse (+ ALEXA FLUOR 594-conjugated streptavidin, ab272189, Abcam) ALEXA FLUOR 488-conjugated anti-rabbit	1:200 1:200	Ab64255, Abcam, Paris, France Ab150077, Abcam, Paris, France	60 min (RT)

RT: room temperature.

### Testicular sperm extraction and sperm analyses

Because of the low production of spermatozoa in vitro, testicular fragments were pooled at the end of cultures. For each group (control, VCR, CYP or VCR +CYP), 24 fragments from 6 different testes were pooled. In total, 96 fragments from 24 different 6 d*pp* testes were in vitro cultured for sperm analyses (Supplementary Fig. 1). Tissues pooled into 1 mL of α-MEM were mechanically dispersed using 27-gauge needles. After a 10-min centrifugation at 300 ×g, pellets containing testicular cells were recovered. Cells were then either smeared onto Superfrost Plus slides (Thermo Fischer Scientific) for the evaluation of sperm yield and the analysis of sperm morphology after Shorr staining (Merck, Darmstadt, Germany) or fixed with methanol (30 min, − 20 °C) and then spread onto glass slides for TUNEL assays. Briefly, after a 2-min permeabilization in acetone, TUNEL assays were performed using the In Situ Cell Death Detection kit POD (Roche, Mannheim, Germany) for 1 h at 37 °C, following manufacturer’s instructions. Slides were mounted in Vectashield with DAPI. Positive controls were performed by incubating spermatozoa with 50 μg/mL DNase I (Sigma-Aldrich) for 15 min at 37 °C before TUNEL assays and negative controls were carried out by omitting the enzyme. Sperm DNA fragmentation was analysed with an Axioskop fluorescence microscope equipped with an AxioCam 503 camera (Carl Zeiss SAS, Marly-le-Roi, France). For each group, hundred spermatozoa (from a pool of 6 in vitro cultured testes) were examined.

### Statistical analyses

Data are presented as mean and standard error of the mean (mean ± SEM) for quantitative variables or number and percentage for qualitative variables. Statistical analyses were carried out with the GraphPad Prism v6.0 software (GraphPad Software Inc., La Jolla, CA, USA). The non-parametric Kruskall-Wallis test followed by Dunn’s post hoc tests were performed to compare VCR, CYP and VCR+CYP groups with control group. The non-parametric Mann–Whitney test was used to compare VCR to VCR+CYP and CYP to VCR+CYP in order to highlight a potential additive gonadotoxic effect of VCR+CYP treatment relative to VCR or CYP alone. Data obtained for TUNEL assays were compared using the χ2 test. A *P *value < 0.05 was considered statistically significant.

## Supplementary Information

Supplementary Information.

## Data Availability

All data generated or analysed during this study are included in this article and Supplementary Fig. 1.
