# Supplementary material for: Achievement of complete in vitro spermatogenesis in testicular tissues from prepubertal mice exposed to mono- or polychemotherapy
Source: Sci Rep. 2022 May 6;12:7407. doi: 10.1038/s41598-022-11286-6 (PMC9076692; doi:10.1038/s41598-022-11286-6)
Supplement: Supplementary file 1 — Supplementary Information. [file 41598_2022_11286_MOESM1_ESM.docx]

**Running title: *In vitro* maturation of mouse testes after chemotherapy**

**Title:** **Achievement of complete *in vitro* spermatogenesis in testicular tissues from prepubertal mice exposed to mono- or polychemotherapy**

Marion Delessard, Laura Stalin, Aurélie Rives-Feraille, Laura Moutard, Justine Saulnier, Ludovic Dumont, Nathalie Rives^†^ and Christine Rondanino^†^*

Normandie Univ, UNIROUEN, INSERM, U1239, Rouen University Hospital, Team Adrenal and Gonadal Pathophysiology, Laboratory of Neuroendocrine Endocrine and Germinal Differentiation and Communication, F 76000 Rouen, France

***Corresponding author:** Dr Christine Rondanino, Ph.D.; email: christine.rondanino@univ-rouen.fr; phone: +33 2 35 14 82 94.

^†^Nathalie Rives and Christine Rondanino contributed equally to the study

**Key words**: chemotherapy, prepubertal exposure, fertility restoration, *in vitro* spermatogenesis, vincristine, cyclophosphamide

**Supplementary figure 1: Flow chart of the study design**

This experimental study investigated the effects of exposure of prepubertal mice to chemotherapy (VCR, CYP, VCR+CYP) on the first wave of *in vitro* spermatogenesis. A total of 40 prepubertal CD-1 mice aged 3 d*pp* received either an intraperitoneal injection of vehicle (control), 100 µg/kg VCR, 15 mg/kg CYP or 100 µg/kg VCR + 15 mg/kg CYP. Testicular tissues from 6 d*pp* mice (n=16 mice, 32 testes) were directly analyzed to investigate the early effects of chemotherapy on testis-to-body weight ratios, spermatogonia-to-Sertoli cells ratios, tissue structural integrity, germ cell content, intratubular cell proliferation, apoptosis and DNA DSBs before *in vitro* culture. In addition, 48 testes were cultured *in vitro* to study the ability of spermatogonia to differentiate during the first wave of spermatogenesis (n=24 mice): the progression of *in vitro* spermatogenesis, germ cell content, intratubular cell proliferation, apoptosis and DNA DSBs were investigated following 30 days of *in vitro* maturation. Finally, 24 other testes were cultured to determine the sperm yield and assess sperm DNA fragmentation.
